# Introductory Addresses

**Published:** 1848-08

**Authors:** 


*Introductory Addresses.*—Since our former notice of Introductory Addresses in the last volume of this Journal, a few additions to our list have been received—to wit:—*Address delivered at the Castleton Medical College by Joseph Perkins, M. D., Prof. of Mat. Medica and Obstetrics.* Prof. Perkins may be ranked among the class of veteran teachers, and the character of this address is what would be expected from a writer of his ability and experience.

*Address delivered at the Castleton Medical College by Wm. Sweetser, M. D., Prof. of the Theory and Practice of Medicine.*

*Valedictory Address by Henry Gibbons, M. D., Prof. Institutes and Practice of Medicine, at the Philadelphia College of Medicine.*

We tender our thanks to the authors for remembering us, and shall always be happy to receive similar favors. We should do them the justice to give our readers extracts, were it not that we had considered the last year's crop disposed of, and the next harvest of introductories is so nigh at hand.

